# An Evaluation of Real-world Smart Sock–Based Temperature Monitoring Data as a Physiological Indicator of Early Diabetic Foot Injury: Case-Control Study

**DOI:** 10.2196/31870

**Published:** 2022-04-01

**Authors:** Alexander M Reyzelman, Chia-Ding Shih, Gregory Tovmassian, Mohan Nathan, Ran Ma, Henk Jan Scholten, Kara Malhotra, David G Armstrong

**Affiliations:** 1 California School of Podiatric Medicine Samuel Merritt University San Francisco, CA United States; 2 Siren Care Inc San Francisco, CA United States; 3 Department of Surgery Southwestern Academic Limb Salvage Alliance Keck School of Medicine of University of Southern California Los Angeles, CA United States

**Keywords:** diabetes, diabetic foot ulcer, temperature monitoring, Charcot foot, digital health, wearable, neuropathy, remote patient monitoring, foot ulcer, monitoring device, patient monitoring

## Abstract

**Background:**

Lower extremity complications of diabetes represent major health care complications both in terms of cost and impact to quality of life for patients with diabetic peripheral neuropathy. Temperature monitoring has been shown in previous studies to provide a useful signal of inflammation that may indicate the early presence of a foot injury.

**Objective:**

In this study, we evaluated the temperature data for patients that presented with a diabetic foot injury while using a sock-based remote temperature monitoring device.

**Methods:**

The study abstracted data from patients who were enrolled in a remote temperature monitoring program (2020-2021) using a smart sock (Siren Care). In the study cohort, a total of 5 participants with a diabetes-related lower extremity injury during the study period were identified. In the second comparison cohort, a total of 26 patients met the criteria for monitoring by the same methods but did not present with a diabetes-related podiatric lower extremity injury during the same period. The 15-day temperature differential between 6 defined locations on each foot was the primary outcome measure among subjects who presented with a diagnosed foot injury. Paired *t* tests were used to compare the differences between the two groups.

**Results:**

A significant difference in temperature differential (temperature measured in °F) was observed in the group that presented with a podiatric injury over the course of evaluation versus the comparator group that did not present with a podiatric injury. The average difference from all 6 measured points was 1.4 °F between the injury group (mean 3.6, SD 3.0) and the comparator group (mean 2.2, SD 2.5, *t*=–71.4, *df*=39; *P*<.001).

**Conclusions:**

The results of this study suggest temperature monitoring in a sock form factor could be used to predict a developing foot injury. The continuous temperature monitoring system employed has implications for further algorithm development to enable early detection. The study was limited by a nonrandomized, observational design with limited injuries present in the study period. We look forward to further studies that will refine the predictive potential and confirm or refute the current promising data.

## Introduction

Diabetic foot ulcers (DFUs) represent a major challenge for the health care system as both a major contributor of health care cost and the greatest single contributor to lower limb amputation [1]. DFUs are estimated to cost public and private payers in the United States US $9-13 billion in addition to the costs associated with diabetes itself [2].

To curtail the devastating ramifications of DFUs, greater efforts have been made toward prevention. One method of early detection that has been studied is the use of temperature monitoring, which has been shown to be a surrogate marker. The elevation of temperature is from inflammation that may be potentially due to and a precursor to tissue injury. The predictive potential of temperature monitoring to detect ulceration was first proposed in 1994 by measuring mean plantar foot temperature by Benbow et al [3]. The potential for foot temperature monitoring was further advanced by Armstrong et al [4], who demonstrated the potential of measuring 6 sites on each foot, with a temperature differential of >4 °F signaling an inflammatory response significant enough to either warrant a change in behavior or seek medical attention. The authors concluded high temperature gradients between feet may predict the onset of neuropathic ulceration, and self-monitoring may reduce the risk of ulceration. Their results showed patients using temperature monitoring were one-third less likely to develop an ulcer compared with the standard therapy group. Armstrong’s study and others have led to the inclusion of temperature monitoring in the guidelines for management of the diabetic foot [5].

The introduction of new technologies for the remote temperature monitoring of patients with diabetes and neuropathy at risk of ulcer formation suggests more patients may be able to use temperature monitoring in their daily lives. One such technology, Siren Socks (Siren Care), is a smart sock worn by patients; it has a regular connection to the cloud for the capture and sharing of temperature data with health care professionals. Siren Socks are available for patients under the supervision of a physician. Reyzelman et al [6] first evaluated Siren Socks in a 35-patient study and found the temperatures measured by the stand-alone sensors were within 0.36 °F of the reference standard. Patients reported the socks were comfortable and easy to use, ranking them at a median score of 9 and 10 on a 10-point scale for comfort and ease of use, respectively.

In this study, we review the actual temperature recordings and real-world monitoring data from patients using remote temperature monitoring. The purpose of this study is to investigate any significant difference of foot temperature prior to the presentation of a foot injury as confirmed by a medical diagnosis.

## Methods

### Study Design

Patients were retrospectively reviewed in the Siren Care data registry between December 1, 2020, and April 15, 2021. Inclusion criteria consisted of patients who had greater than 50 days of wear of the Siren Socks temperature monitoring device during the study period. To be eligible to use Siren Socks, patients needed to have a diagnosis of peripheral neuropathy. The study cohort included patients in the database who had a diagnosed lower extremity injury that presented itself during the study period. The 15-day period prior to the diagnosis of an injury was reviewed, and a total of 900 minutes of temperature monitoring were analyzed during the period. For the control cohort, patients meeting the same criteria of wear (ie, diagnosed with peripheral neuropathy) without reported foot injuries in the study period were selected. For the control cohort, the temperature monitoring data for a randomly selected 900 minutes over a randomly selected 15 days were chosen for comparison.

The temperature data were reviewed retrospectively for a 15-day period before the presentation of an injury to a medical professional. For comparison, a similar 15-day period of temperature monitoring was reviewed for other patients who wore the Siren Socks but did not present with an injury in the study period.

### Description of Temperature Monitoring Workflow

Patients in both cohorts were prescribed remote patient monitoring socks by their podiatrist. The patients in both cohorts were under the care of a podiatrist who directed a licensed practical nurse (LPN) to regularly monitor these patients based on temperature monitoring data, and to escalate any identified issues to their attention for possible clinical follow-up and intervention. Each patient had continuous measurements of temperature taken at 6 points on each foot (hallux, heel, arch, metatarsal 1, metatarsal 3, and metatarsal 5). The temperature is measured automatically throughout the day. The socks turn on when worn and turn off automatically when no longer worn. No charging is required, and data transmission does not require a smartphone. A hub is plugged into the wall for data transmission, and monitoring data is also stored on the socks to allow for monitoring when away from home.

**Figure 1:**
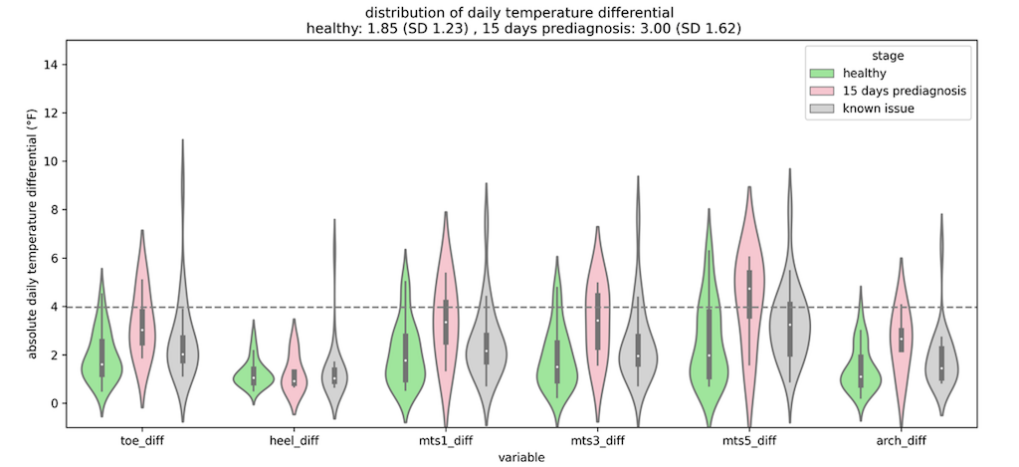
The temperature difference of a patient who developed a foot ulcer. The green plot reveals the baseline measurements, the red plot shows the 15 days before the ulcer was diagnosed, and the grey plot shows the active ulcer period.

**Figure 2:**
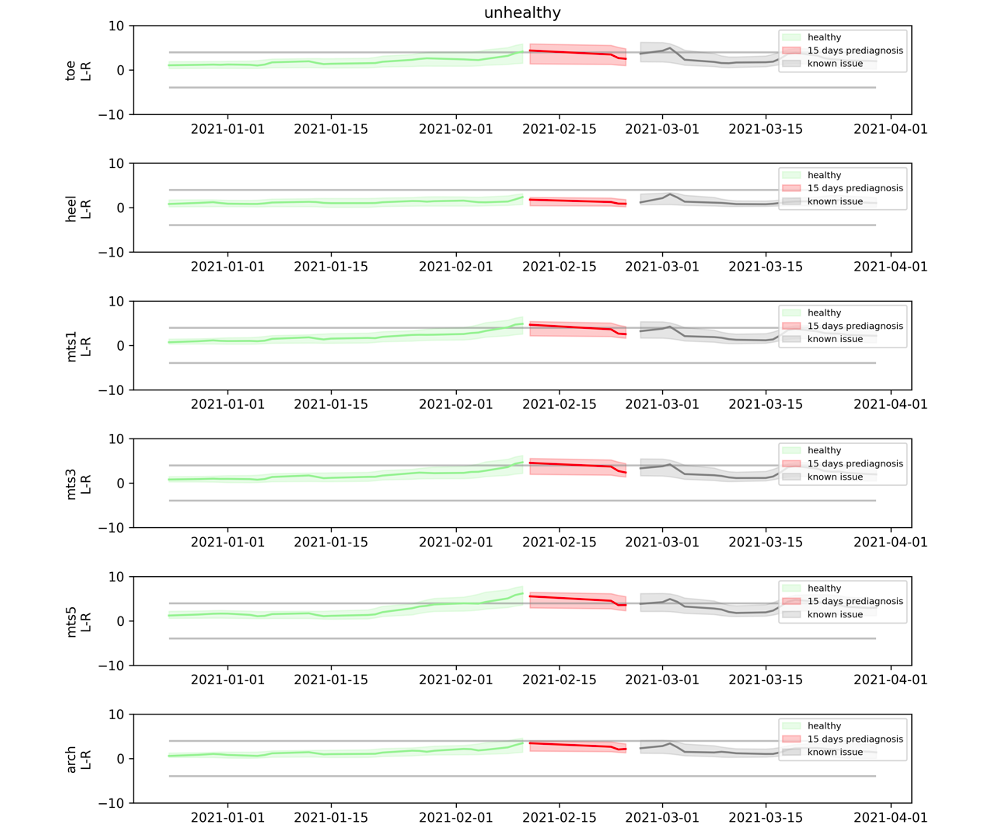
The temperature differentials measured over the study period for a patient who developed an ulcer at the right fifth metatarsal (mts) location. The green plot represents the baseline period, the red plot is the period 15 days prior to the ulcer presenting, and the grey plot is the period after the issue was diagnosed by a medical professional. Changes in temperature trends are noticeable even before the 15-day period, suggesting earlier detection of foot ulcers might be possible.

Data are collected at each point in the foot, and the temperature differential between each right and left point is computed each minute. Finally, the daily average temperature differential is computed for each area on the foot and each patient. Figure 1 and Figure 2 are examples of the data capture for study patients.

### Description of Follow-up Routine

The data from the remote patient monitoring device were reviewed by LPNs under the supervision of a podiatrist. Any temperature differentials greater than 4 °F sent an alert to the LPN that required follow-up via a phone call to the patient. In each case, the sustained level of temperature rise with the possible presentation of an injury caused the patient to be referred to the clinic for evaluation. In the case of the control cohort, the temperature was monitored using the same method used for the study cohort, and the same alert criteria for temperature differential and follow-up routine were used.

### Description of Outcome Measures

The primary outcome measure is the difference in temperature between two points on the feet of a patient.

### Statistical Analysis

**Figure 3:**
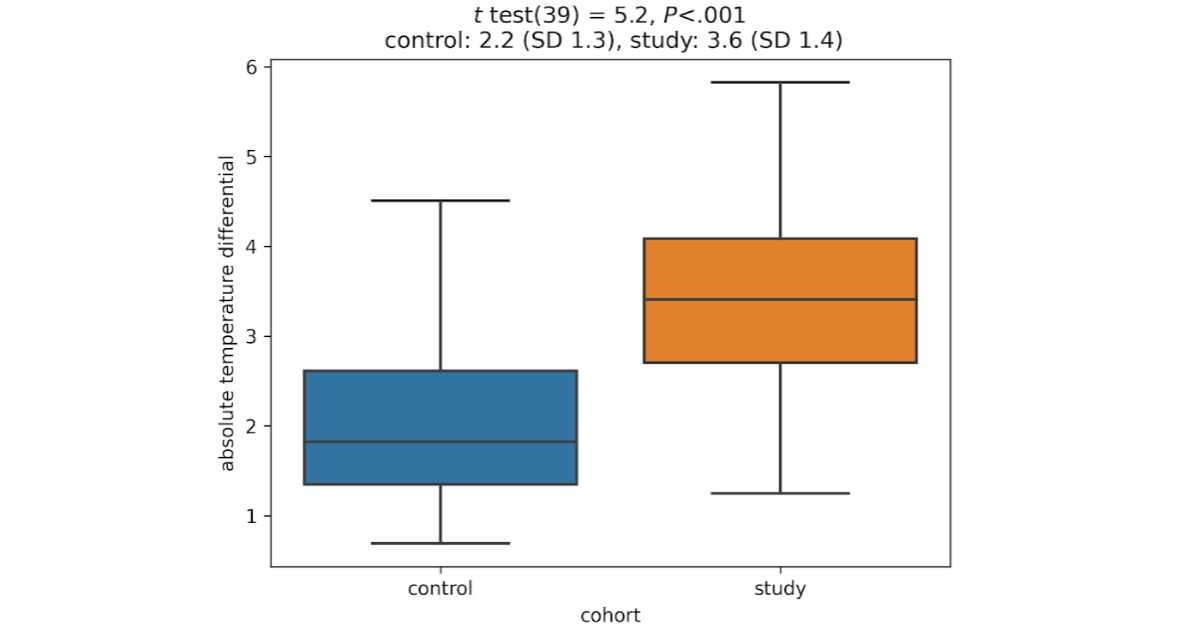
Comparison of the average temperature differential between two cohorts.

A Welch *t* test was applied to determine that the mean temperature deviation in the study cohort was significantly higher than the control cohort (Figure 3) (*P*<.001). The analysis was performed using SciPy v1.6.3 programmed in Python (Python Software Foundation).

### Ethics Approval

This study was approved by the WCG IRB (study number 12843666). If an individual wished to participate in the study, they were informed about the study objectives, and they could consent through a mobile app or over the phone after having started using the socks.

## Results

A review of the relevant patient demographics is presented in Table 1. None of these differences are considered clinically relevant in terms of temperature data observations. During the observation period, a total of 5 patients presented with lower extremity injury with a temperature differential of 4 °F compared to the contralateral foot. The profile of each injury is listed in Table 2.

**Table 1:** Patient demographics.

Criteria		Study cohort (N=5)	Control cohort (N=26)
Average age (years)		66.0	70.4
**Sex, n**			
	Female	1	9
	Male	4	17
**Additional diagnosis, n (%)**			
	Diabetes	3 (60)	24 (92)
	Peripheral artery disease	2 (40)	5 (19)

**Table 2:** Study cohort.

Injury type	Age (years)	Patient history	Clinical notes
Charcot arthropathy	68	Neuropathy, type 1 diabetes	5-6 days of hotspots. Patient saw provider in clinic for diagnosis of early onset Charcot. Treatment: stay off of foot, use CAM^a^ walker, x-rays of right foot.
Ulcer	75	Type 2 diabetes	New diagnosis added: traumatic blister of right hallux—right blister (nonthermal), right lesser toe(s), initial encounter. Crest pads added to shoes.
Osteomyelitis	61	Peripheral artery disease	Persistent hotspots at all 6 foot locations, patient hospitalized for infection symptoms. Osteomyelitis diagnosed with subsequent angioplasty and stent placement.
Fifth metatarsal head ulcer	48	Type 2 diabetes, history of ulcers	Right fifth metatarsal diagnosis changed to ulcer during provider visit. Ulcer was debrided. Go to his cast boot. Continue Siren Socks. Antibiotic ointment to the wound.
Blood clot	76	Peripheral artery disease	Patient began alerting with temperature differential in entire right foot. Patient reported thigh, knee, calf, and foot are swollen. Provider discussed and advised to go to emergency room where deep vein thrombosis was diagnosed and treated.

^a^CAM: controlled ankle movement.

A mean significant temperature increase of 3.59 °F (SD 1.42) was observed in the study cohort in the 15 days preceding an injury confirmed by physical medical examination. The control cohort had a mean temperature differential of 2.20 °F (SD 1.31) during a 15-day comparative period. The difference in means between the two cohorts was 1.4 °F (95% CI 0.859-1.20). Of note, the *P* value between the two cohorts was <.001, demonstrating statistical significance between the two cohorts as to the level of temperature differential.

## Discussion

### Principal Findings

The prevalence of DFUs and the extent of the clinical complications suggest new methods must be explored. Though temperature monitoring on an episodic basis has been previously described in the literature, this study appears to be the first to use a continuous temperature monitoring device in a real-world environment. The goal of this evaluation was to determine how temperature data would be different, particularly as an early warning indicator, for those patients presenting with a diagnosed foot injury. Previous studies have demonstrated the inflammatory response to injury does lead to a measurable increase in temperature [7,8].

The results of this study suggest that an inflammatory signal is seen with temperature monitoring when there is an injury. Of note, the presence of the temperature difference in the 15-day period prior to the patient presenting and the injury being assessed by a clinician is of particular interest in terms of potential impact on clinical practice. If at-risk patients routinely used a remote temperature monitoring device, it might be possible to identify risks and intervene sooner than standard practice currently allows. In addition to observing the absolute temperature differential, the creation of a continuous temperature monitoring device offers new possibilities to establish a baseline level of variation for a particular patient. The potential exists for significant temperature data to be used to create algorithms to better predict the early formation of podiatric injuries.

### Study Limitations

The study has several limitations. There was a small number of injuries, which limited the study population. As the overall number of patients using a remote patient monitoring solution grows, there will likely be a much larger number of cases where a foot injury diagnosis is made. The study was limited to observations made in a 135-day period. The 15-day period was chosen for evaluation, but data certainly suggested temperature differentials of longer periods are of interest. Further study is needed with greater numbers of patients to establish the optimal early detection period.

### Comparison With Prior Work

Several studies have been published that evaluate the role of temperature monitoring in the detection or possible prevention of DFUs. Of note, this study appears to be the first to evaluate a continuous temperature monitoring device in patients who did and did not experience a podiatric injury, with both groups providing continuous temperature monitoring data. In an evaluation of an episodic temperature monitoring device, the threshold of 4 °F was used to predict 97% of observed ulcers in a study of patients at risk of recurrent DFUs, but the false-positive rate was 57% [9]. Raising the temperature differential threshold reduced sensitivity but also reduced the false-positive rate. Another study looked at the validity of a specific 4 °F threshold for ulceration detection and postulated daily variations could influence outcomes [10]. The authors suggested future research should identify ways to use continuous monitoring sensors to further define individual thresholds.

### Conclusion

The results of this study suggest temperature monitoring using a sock form factor may be a predictor of a developing foot injury. The study cohort had a mean foot temperature differential that was significantly different from that of the control cohort. The ability to review continuous temperature data in a 15-day period prior to a recognized problem showed that temperature differences beyond expected baseline variation were observed. The predictive value of these temperature data suggests patients and providers may become aware and engage earlier to address an issue before it progresses to a more serious level.

The value of temperature monitoring has been demonstrated in controlled studies in the past, but limited real-world data exist of its use in clinical practice. This study showed a statistically significant difference in the continuous temperature monitoring differential of patients who presented with a podiatric injury in the 15-day period prior to seeing a health care professional. Further study is warranted in larger patient groups over a longer follow-up period to better understand the predictive power of temperature monitoring for earlier detection of foot injury in patients with neuropathy and diabetes.

## Abbreviations

DFU: diabetic foot ulcer
LPN: licensed practical nurse
